# P01.34. Use of plant bioassays in homeopathic basic research: a systematic review

**DOI:** 10.1186/1472-6882-12-S1-P34

**Published:** 2012-06-12

**Authors:** S Baumgartner, L Betti, P Heusser, T Jäger, V Majewsky, U Wolf

**Affiliations:** 1 University of Bern, Bern, Switzerland; 2 University of Bologna, Bologna, Italy; 3 University of Witten-Herdecke, Herdecke, Germany; 4 University of Bern, Institute of Complementary Medicine KIKOM, Bern, Switzerland

## Purpose

Experimental research on the effects of treatments with homeopathic preparations on plants was last reviewed in 1990. The objective was to compile a systematic literature review on plant bioassays in homeopathic basic research using predefined criteria.

## Methods

A literature search was carried out on publications that reported experiments with homeopathic preparations on whole plants, seeds, plant parts and cells from 1920 to 2010, in healthy, abiotically or biotically stressed conditions. Outcomes had to be measured by established state-of-the-art procedures and statistically evaluated. Using a Manuscript Information Score (MIS), publications were identified that provided sufficient information for proper interpretation (MIS > 5). Further evaluation focused on the use of adequate controls to investigate specific effects of homeopathic preparations and on the use of systematic negative control experiments to ensure proper system performance.

## Results

A total of 157 publications with plants were identified. The 157 publications described a total of 167 experimental studies. Eighty-four studies included statistics and 48 had a MIS > 5 allowing proper interpretation. Twenty-nine studies were identified with adequate controls to identify specific effects of homeopathic preparations, reporting significant effects of decimal and centesimal homeopathic potencies, including dilution levels beyond Avogadro’s number. Studies that tested series of consecutive potency levels reported a non-linear and discontinuous relation between effect and potency level. There were many individual studies with diverse methods and very few replication trials. Ten studies reported use of systematic negative control experiments, yielding evidence for the stability of the experimental set-up.

## Conclusion

Plant models appear to be a useful approach to investigate basic research questions on homeopathic preparations, but more independent replication trials are needed. Systematic negative control experiments should be implemented on a routine basis to exclude false-positive results.

